# Constancy and trade-offs in the neuroanatomical and metabolic design of the cerebral cortex

**DOI:** 10.3389/fncir.2014.00009

**Published:** 2014-02-11

**Authors:** Jan Karbowski

**Affiliations:** ^1^Nalecz Institute of Biocybernetics and Biomedical Engineering, Polish Academy of SciencesWarsaw, Poland; ^2^Department of Mathematics, Informatics and Mechanics, Institute of Applied Mathematics and Mechanics, University of WarsawWarsaw, Poland; ^3^Division of BiologyCaltech, Pasadena, CA, USA

**Keywords:** cerebral cortex, conservation, connectivity, metabolism, capillary, constraints, allometry, evolutionary design

## Abstract

Mammalian brains span about four orders of magnitude in cortical volume and have to operate in different environments that require diverse behavioral skills. Despite these geometric and behavioral diversities, the examination of cerebral cortex across species reveals that it contains a substantial number of conserved characteristics that are associated with neuroanatomy and metabolism, i.e., with neuronal connectivity and function. Some of these cortical constants or invariants have been known for a long time but not sufficiently appreciated, and others were only recently discovered. The focus of this review is to present the cortical invariants and discuss their role in the efficient information processing. Global conservation in neuroanatomy and metabolism, as well as their correlated regional and developmental variability suggest that these two parallel systems are mutually coupled. It is argued that energetic constraint on cortical organization can be strong if cerebral blood supplied is either below or above a certain level, and it is rather soft otherwise. Moreover, because maximization or minimization of parameters associated with cortical connectivity, function and cost often leads to conflicts in design, it is argued that the architecture of the cerebral cortex is a result of structural and functional compromises.

## Introduction

Bigger mammals tend to have bigger brains (Jerison, 1973; Haug, 1987; Hofman, 1988; Allman, 1999). There exists five orders of magnitude difference in brain volume between the smallest mammal (Etruscan pygmy shrew with 0.04 g brain; McNab and Eisenberg, 1989) and the largest (sperm whale with ~10 kg brain; Haug, 1987). Despite such a big span in size, brains of different species share common structures with similar properties (Hofman, 1988, 1989; Finlay and Darlington, 1995; Barton and Harvey, 2000; Clark et al., 2001; De Winter and Oxnard, 2001). This similarity in structure and function is presumably an indication of the same basic mechanism governing the developmental process in all mammals (Striedter, 2005). Some departures from a common plan can be explained by interactions of genetic (molecular) and environmental factors(Krubitzer, 1995).

Cerebral cortex, which is responsible for processing sensory, behavioral, and cognitive informations (Kandel et al., 1991), can also differ vastly in mass and geometric dimensions across mammals (Hofman, 1988, 1989), and yet it contains neuroanatomical characteristics that are roughly independent of brain size (Braitenberg and Schüz, 1988; DeFelipe et al., 2002). The first purpose of this review is to systematically present and discuss these structural constants or invariants, and to show that a similar remarkable conservation is also associated with cortical metabolism and its underlying hemodynamics and microvasculature.

It seems that the presence of invariants in the structure and dynamics of cortical networks has not been fully appreciated in the neuroscience community. There exists a vast literature focused much more on differences than on similarities in the cerebral structure and morphology in order to explain behavioral and cognitive diversities among mammals (e.g., Krubitzer, 1995; Herculano-Houzel et al., 2008; Rakic, 2009; Herculano-Houzel, 2011a). However, cortical conservation is an important empirical fact (Braitenberg and Schüz, 1998; DeFelipe et al., 2002; Karbowski, 2003; Douglas and Martin, 2004), which deserves more analysis. Such analysis may provide clues regarding basic principles of anatomical and functional organization of the cerebral cortex. This knowledge may be useful in expanding our general understanding of brain evolution and development. It is rather unlikely that cortical invariants are the result of an evolutionary accident. Instead, their existence suggests a certain universality in cortical design. In this context, the challenging questions are: (1) what is the cause of cortical invariants, (2) how are they inter-related, and (3) what are the possible benefits they might bring for efficient functioning of the brain?

The second aim of this review is to show that cortical neuroanatomy and metabolism are mutually interrelated, in such a way that cortical invariants lead to various functional and energetic trade-offs. Related to this is the issue of an extent to which metabolism restricts cortical architecture, which will be also briefly discussed.

That energy should play some role in constraining the evolution of brain size (Martin, 1981; Isler and van Schaik, 2006; Navarrete et al., 2011) and neural information processing (Laughlin et al., 1998; Balasubramanian et al., 2001; Niven and Laughlin, 2008) is generally accepted. This is mainly because cerebral tissue is metabolically expensive (Aiello and Wheeler, 1995; Attwell and Laughlin, 2001; Karbowski, 2007). This cerebral expensiveness is particularly visible in the allometric scaling of brain energetics across species: energy consumption of the brain grows with brain size faster than a corresponding energy use of the whole body increase with body size (Karbowski, 2007). For this reason, it may seem that cortical design should be related to the economy of energy expenditure, similar to the design of circulatory system in animals (Weibel et al., 1991).

## Neuroanatomical invariants

One has to realize that neurobiological constancy does not mean an exact mathematical constancy. Rather, it is meant statistically. In biology there is always some variability of “constant” parameters across individuals or species, and therefore we usually take average values for statistical analysis across species. In this study, by constant or invariant parameters we mean characteristics whose average values do not change significantly with brain size. Most cortical invariants are associated with synapses and neuronal wiring.

### Synaptic size

**Figure 1:**
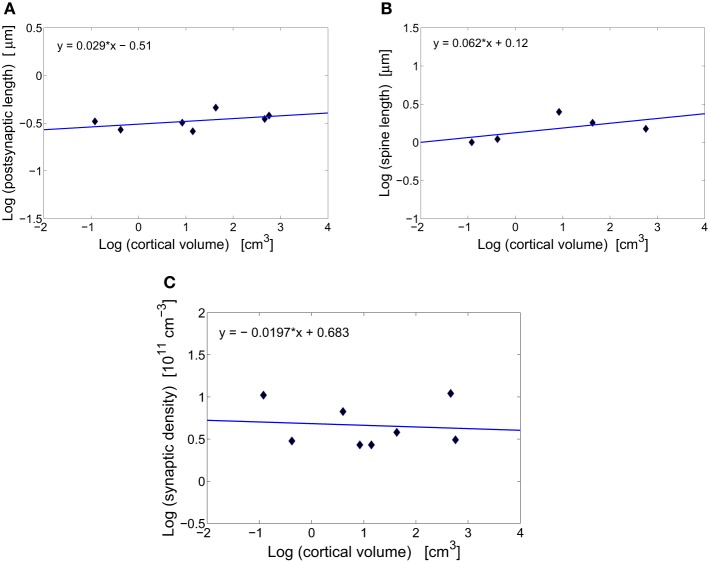
**Scaling of synaptic characteristics with cortical volume**. **(A)** Conservation of postsynaptic density length of excitatory synapses across mammals. The log–log fit to the data points yields a scaling exponent close to zero (*y* = 0.029x − 0.51), with non-significant moderate correlations (*r* = 0.480, *p* = 0.275). **(B)** Conservation of spine length of excitatory synapses across mammals. The scaling exponent is close to zero (*y* = 0.062x+ 0.12) with non-significant moderate correlations (*r* = 0.576, *p* = 0.310). **(C)** Conservation of the total synaptic density across mammals. The log–log fit gives a scaling exponent close to zero (*y* = −0.020x + 0.683) with non-significant weak correlations (*r* = −0.099, *p* = 0.815).

Synapses in the cerebral cortex play a special role. They are thought to encode, through some structural changes, information that is vital for animal's living and survival (Kasai et al., 2010; Bourne and Harris, 2011). Without these plastic changes animals would be unable to learn and remember events occurring in the environments. Because of the plasticity, synaptic sizes vary widely and can differ even by a factor of ~30 within the same cortical area of an individual (Loewenstein et al., 2011). However, in spite of this geometric variability the average size of excitatory synapses stays roughly constant across different species (Table 1, Figures 1A,B). Specifically, the length of postsynaptic density is essentially independent of cortical volume, and is in the range 0.27–0.46 μm (Table 1, Figure 1A). What is even more interesting, this average length does not seem to change much during postnatal development or even aging, at least in primates (Zecevic and Rakic, 1991; Huttenlocher and Dabholkar, 1997; Peters et al., 2008). The spine length of excitatory synapses (combined length of spine neck and head) is also conserved across mammals (Table 1, Figure 1B). The invariance of the average synaptic dimensions indicates that structural synaptic machinery (different receptors) and design is very similar among mammals.

**Table 1:** **Synaptic and wiring characteristics for mammalian cerebral cortex**.

**Species**	**Synaptic density ρ_s_ (10^11^cm^−3^)**	**Excitatory synapses (%)**	**Postsynaptic density length (μm)**	**Spine density on dendrite (μm^−1^)**	**Spine length (μm)**	**Dendrite diameter basal (μm)**
Mouse	10.5 ± 2.9^a^	89^b^	0.33 ± 0.02^a^	1.9 ± 0.4^a^	1.0 ± 0.0^c^	0.9^a^
Rat	3.0 ± 0.1^d^	89^b^	0.27 ± 0.00^d^	3.4 ± 1.1^c^	1.1 ± 0.0^c^	0.6 ± 0.1^c^
Echidna	2.7 ± 0.2^e^	72^f^	0.32 ± 0.12^e^	1.2 ± 0.4^e^	2.5^e^	1.0 ± 0.2^e^
Rabbit	6.7^g^	−	−	0.7 ± 0.1^h^	−	−
Cat	2.7 ± 0.2^i^	84^j^	0.26 ± 0.01^k^	0.7 ± 0.1^j^	−	1.0^l^
Macaque	3.8 ± 0.4^m^	75^n^	0.46 ± 0.02^n^	1.5 ± 0.3^o^	1.8 ± 0.1^c^	1.4^c^
Dolphin	11.0 ± 2.0^p^	81^p^	0.35 ± 0.12^p^	−	−	−
Human	3.1 ± 0.3^q^	89^b^	0.38 ± 0.04^r^	1.2 ± 0.2^s^	1.5 ± 0.1^s^	0.7 ± 0.1^s^

*Synaptic density includes both excitatory and inhibitory synapses and refers to visual cortex in all animals except echidna (somatosensory cortex). Data for other parameters come from different cortical regions. Postsynaptic density length, spine density, and spine length all correspond to excitatory (asymmetric) synapses. Basal dendrite diameter refers to pyramidal cells only*.

References:

a Braitenberg and Schüz (1998);

b DeFelipe et al. (2002);

c Escobar et al. (2008);

d Blue and Parnavelas (1983);

e Hassiotis et al. (2003);

f Hassiotis et al. (2005);

g Vrensen et al. (1977);

h Mathers (1979);

i Winfield (1981);

j Binzegger et al. (2004);

k Cragg (1975);

l Mainen and Sejnowski (1996);

m Bourgeois and Rakic (1993);

n Zecevic and Rakic (1991);

o Elston et al. (2011),

p Glezer and Morgane (1990);

q Huttenlocher and Dabholkar (1997);

r Scheff et al. (2001);

s *Benavides-Piccione et al. (2013)*.

### Synaptic density

Total number of excitatory and inhibitory synapses per unit cortical volume or synaptic density changes non-monotonically during animal's lifetime, taking maximal values often early in the development (Winfield, 1981; Zecevic and Rakic, 1991; Bourgeois and Rakic, 1993; Huttenlocher and Dabholkar, 1997). However, at adulthood synaptic density stabilizes and its mean value (~ 5· 10^11^ cm^−3^) is approximately independent of brain size (Table 1, Figure 1C). There exists some variability across mammals and cortical areas, but it does not depend systematically on cortical volume (Figure 1C). This may suggest that neural plasticity can have an additional role, namely to find optimal and species independent values for synaptic density and size that are needed for efficient and universal cerebral function (Chklovskii, 2004).

### Ratio of excitatory to inhibitory synapses

A related quantity to synaptic density is the ratio of the number of excitatory to inhibitory synapses (i.e., the ratio of asymmetric to symmetric synapses, respectively). Data for adult mammals of different sizes show that this ratio is conserved, and excitatory synapses comprise about 80–90% of all synapses in the cerebral cortex (DeFelipe et al., 2002; Table 1). The degree of this conservation is remarkable (average 0.83 ± 0.03), suggesting that the excitatory to inhibitory ratio is somehow an important parameter for brain operation. One can suspect that the constancy may be necessary for maintaining a dynamical balance in cortical circuits. Probably because of that, neural activities cannot be too high or too low for an efficient cortical function. There have been several experimental reports supporting the notion of a dynamic balance between discharges of cortical excitatory and inhibitory neurons (Haider et al., 2006; Vogels et al., 2011), which might be related to the neuroanatomical constancy of synaptic polarity. Interestingly, this phenomenon and its consequences have been predicted almost two decades ago in a theoretical study (Van Vreeswijk and Sompolinsky, 1996).

### Diameter of unmyelinated axons and dendrites

**Figure 2:**
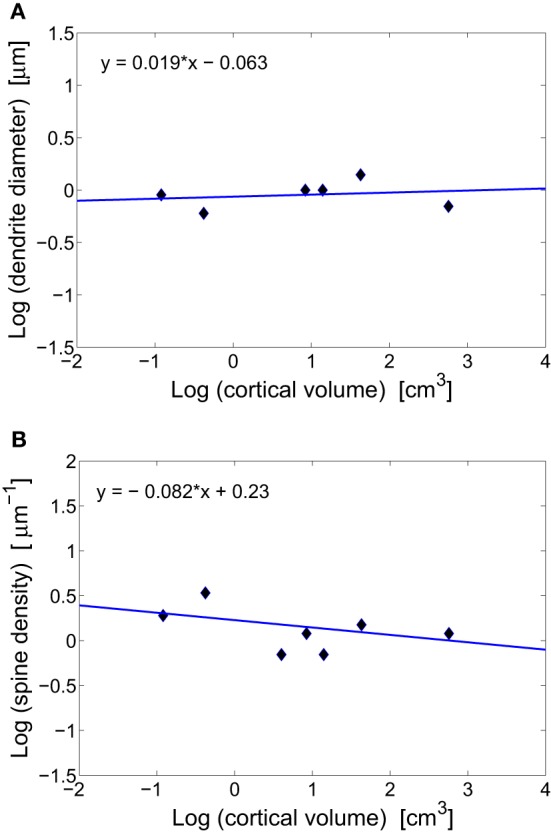
**Scaling of dendritic characteristics of pyramidal cells with cortical volume**. **(A)** Conservation of basal dendrite diameter across species. The log–log fit gives a scaling exponent around zero (*y* = 0.019x − 0.063), with non-significant weak correlations (*r* = 0.202, *p* = 0.702). **(B)** Conservation of spine density on a dendrite across mammals. The log–log fit gives a scaling with a small non-significant exponent (*y* = −0.082x + 0.23, *r* = −0.477, *p* = 0.338).

The vast majority of cortical excitatory synapses are made between presynaptic axonal boutons and postsynaptic dendritic spines (Blue and Parnavelas, 1983; Glezer and Morgane, 1990; Zecevic and Rakic, 1991). It has been suggested that diameters of intra-cortical axons and dendrites do not change systematically with brain size (Braitenberg and Schüz, 1998). The data gathered in Table 1 for diameters of basal dendrites of pyramidal cells in the cortex of different mammals support this prediction (Figure 2A). The issue is slightly different for white matter long-range axons whose average diameters tend to increase weakly with brain volume, mostly due to the thickest 5% (Olivares et al., 2001; Wang et al., 2008). The constancy of the diameter of intracortical wiring may have something to do with the constancy of synaptic parameters, as these two anatomical processes are mutually coupled. That is, synapses need resources and molecular signaling from axons and dendrites to grow and maintain their size. The flow of different molecules, and thus information between these two subsystems is perhaps optimized if both have similar sizes. Indeed, axon diameter of pyramidal cells in gray matter is about 0.2–0.3 μm (Braitenberg and Schüz, 1998), which is close to the average length of synaptic boutons of about 0.25 μm (Escobar et al., 2008). Moreover, the diameter of pyramidal basal dendrites is three to four times larger than that of axons, which is close to the length of dendritic spines (Table 1).

### Fraction of volume occupied by axons and dendrites

Data for volumes of axons and dendrites in the mouse and cat cortex indicate that they are approximately equal, and each of them takes about 1/3 of the cortical volume (Braitenberg and Schüz, 1998; Chklovskii et al., 2002). Given the invariants associated with synaptic, axonal, and dendritic sizes, one can guess that cortical space is compactly filled, and there is not much room for dimensional variability. This may be a result of economical wiring in the cerebral cortex, and competition for space among different neural components (Budd and Kisvarday, 2012; Bullmore and Sporns, 2012). A direct consequence of the wiring volume conservation and the fact that axons are thinner than dendrites is that axons are additionally much longer. This enables neurons to send signals to remote regions to facilitate neural communication. However, the smallness of axonal diameter has also a downside, because it limits the speed of signal propagation (see Discussion).

Another consequence of having invariant synaptic density, dendrite diameter, and the fraction of volume taken by dendrites, is that the density of spines along dendrites of pyramidal cells should be also invariant (this can be easily verified by dividing synaptic density through dendrite fractional volume, both of which are constants). This conclusion agrees well with the data in Table 1 and Figure 2B. From this, it follows that dendritic length and number of synapses per neuron are strongly correlated and should scale with brain size the same way.

### Density of glial cells

Apart from neurons the cortical tissue contains non-neuronal cells called glia. There are different types of glia, each with a specific role in the nervous system. The most numerous are astrocytes (O'Kusky and Colonnier, 1982), which are mainly involved in recycling neurotransmitters (they are mechanically coupled to synapses) and in transport of different nutrients and metabolites between circulating blood and neurons (Nedergaard et al., 2003). Thus astrocytes play an important active regulatory role in cortical metabolism (Magistretti, 2006; Belanger et al., 2011). Less numerous glia, oligodendrocytes and microglia, are involved in providing mechanical support and insulation for neural processes, and in immunological protection, respectively (Kandel et al., 1991).

Studies performed across several mammals indicate that volume density of glia in the cerebral cortex is independent of brain size, and about 2· 10^4^ mm^−3^ (Herculano-Houzel, 2012; Carlo and Stevens, 2013). Considering that synaptic density is also invariant across species, suggests that synapses and glia are mutually coupled not only anatomically but also functionally. Interestingly, in the mouse cortex glia and synapses occupy a similar percentage of cortical volume ~10% (Braitenberg and Schüz, 1998). However, in bigger brains glia could take even more cortical space, because in contrast to synapses, astrocytes increase in size and complexity as brain gets larger (Oberheim et al., 2006). Moreover, the number of glia per neuron in the cortex grows steady with brain size (Oberheim et al., 2006; Herculano-Houzel, 2012), which probably reflects higher metabolic needs of larger brains (Karbowski, 2007). In contrast, number of synapses per glia is independent of brain size, because synaptic and glia densities are invariant across mammals.

## Neuroanatomical parameters weakly dependent on brain size

Cerebral cortex can be also characterized by other parameters, some of which are not precisely conserved across mammals, but nevertheless change with brain size so weakly that can be roughly regarded as almost constant. The most prominent of these are: cortical thickness and elementary cortical module size. The first parameter is associated with the cortical geometry, which is essentially two dimensional, as the cortex grows much more tangentially than radially. For instance, cortical thickness in mouse is 0.8 mm (Braitenberg and Schüz, 1998), whereas in a convoluted elephant cortex it is about 3 mm (Shoshani et al., 2006). These values differ by a modest factor of ~4, despite 10^4^ difference in their cortical mass (Braitenberg and Schüz, 1998; Hakeem et al., 2005).

A similar situation occurs for cortical micro modularity. These micro-modules, known as columns, are thought of as elementary functional units in the cerebral cortex (Mountcastle et al., 1957; Szentagothai, 1978; Buxhoeveden and Casanova, 2002). There is some variability in the column size across species, in the range 200–1000 μm (Buxhoeveden and Casanova, 2002), but it is unclear if it depends systematically on brain size, as there is no universal definition of a column. Another manifestation of cortical modularity is the presence of the so-called patches in the visual cortex (visible with staining methods). Patches have diameters in the range 200–500 μm, and their sizes are roughly independent of brain size (Karbowski, 2003). This approximate constancy or limited variability of elementary cortical modules processing information may have some functional role. However, it should be mentioned that small rodent brains are an exception, as their cortices do not exhibit intra-area patches (Van Hooser et al., 2006; Muir and Douglas, 2011).

## Metabolic and hemodynamic invariants

**Figure 3:**
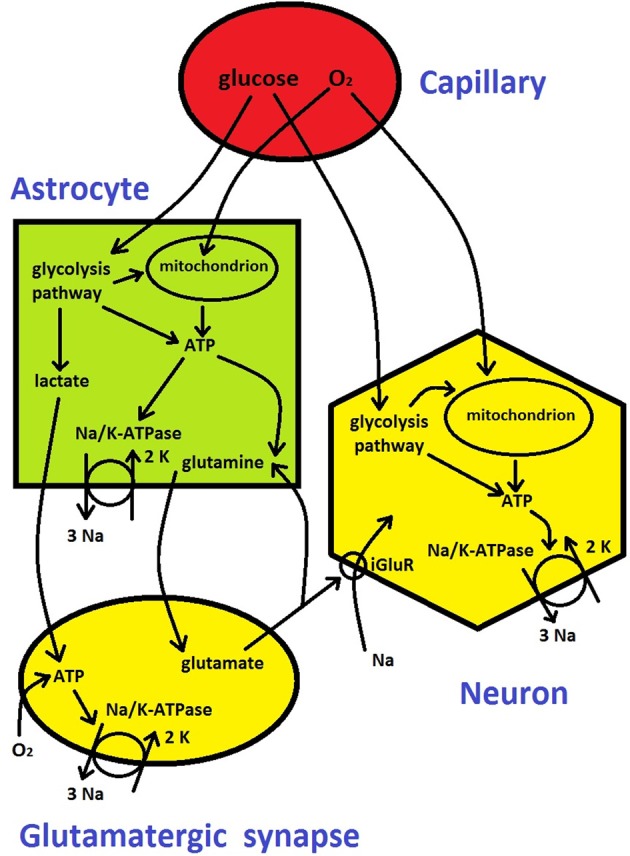
**Metabolic energy flow in the brain (see description in Box 1)**. It is based on diagrams from Attwell et al. (2010) and Belanger et al. (2011).

Brain as a physical object needs energy for its operations (see Box 1, and Figure 3). In a nutshell, without its sufficient supply by cerebral blood flow, neurons and their processes would not grow to nominal sizes, cortical circuits would not be rightly connected, and the whole system would not function properly (Martin et al., 1994). For instance, a sharp reduction in blood supply to the brain for just several seconds leads to a loss of consciousness, and causes irreversible damage to neural structures if blood flow is stopped for few minutes (Raichle, 1983). Given the presence of neuroanatomical invariants, it is interesting to review corresponding invariants associated with cortical energetics and blood circulation.

Box 1 Cerebral blood flow (CBF) in capillaries provides two critically important metabolic substrates, oxygen and glucose, to neurons, synapses, and astrocytes (Figure 3). ATP molecule is an universal “currency” for metabolic activity within cells in all living organisms (Erecinska and Silver, 1989). In the brain tissue ATP is produced in three main ways (Figure 3): either locally through glycolysis pathway and through oxidation of glucose in mitochondria (Attwell et al., 2010), or non-locally through the involvement of lactate (from glycolysis) in astrocytes and its transport and subsequent oxidation in glutamatergic presynaptic terminals (Magistretti, 2006; Belanger et al., 2011). Thus, oxygen and glucose utilizations are strictly related to the metabolic energy (ATP) production, and therefore they are regularly used as measures of cerebral metabolic rate, i.e., CMR_*O2*_ and CMR_glu_, respectively. Depending on their metabolic needs, neurons and synapses during normal function can regulate to some extent the amount of oxygen and glucose supplied by blood either directly or by active signaling to astrocytes, which then modulate capillaries by changing their diameter (Attwell et al., 2010).

The energy released from hydrolysis of ATP is used to maintain the brain in a state that is far from thermodynamic equilibrium, which is mainly characterized by differences in intra- and extra-cellular concentrations of various ions (chiefly Na^+^, K^+^, Cl^−^, and Ca^++^; Ames, 2000). The ionic gradients across membrane are required for keeping negative resting voltages of neurons and for the ability to generate action potentials, which are necessary for inter-neuronal communication.

The majority of the energy goes for pumping ions across neural membrane and for recycling neurotransmitters, in which astrocytes are involved ((Attwell and Laughlin, 2001; Magistretti, 2006); Figure 3). The most important and the most energy demanding is Na^+^/K^+^ pump that maintains Na^+^ and K^+^ concentration gradients through the membrane (Erecinska and Silver, 1989; Ames, 2000). The Na^+^/K^+^ pump performs an electrochemical work by pumping out 3 Na^+^ ions and pumping in 2 K^+^ ions per one pump's cycle, using energy generated by hydrolysis of 1 ATP molecule, and the process is called Na^+^/K^+^-ATPase (Figure 3). In general, neural and synaptic activities cause Na^+^ influx through voltage-gated channels and through ionotropic glutamate receptors (iGluR) at postsynaptic terminals of glutamatergic synapses. Na^+^ influx and corresponding K^+^ efflux counteract the ionic gradients and pull the whole system closer to a thermodynamic equilibrium (concentration of Na^+^ inside neurons and glia is much lower than outside, and the opposite for K^+^). A single action potential has only a marginal influence on the neural gradients, yet, a cumulative effect of many action potentials in a short period of time may significantly alter the gradients because the pump kinetics are slow (Karbowski, 2009). Therefore a continuous electrical and chemical communication between neurons in a non-equilibrium cerebral state requires a restoration of ionic gradients through a permanent pumping, which costs metabolic energy (the rate of ATP generation and hydrolysis). This process makes the mammalian brain one of the most energetic organs (Aiello and Wheeler, 1995; Attwell and Laughlin, 2001; Isler and van Schaik, 2006; Karbowski, 2007; Navarrete et al., 2011). For instance, the adult human brain constitutes of only 1–2% of the total body volume but consumes an excessive amount of 20% of the total body metabolic rate (Mink et al., 1981).

### Global scaling of brain metabolism

Brain metabolic energy consumption grows relatively fast with brain size. Oxygen and glucose utilization per time unit by the whole cerebral tissue increase allometrically with mammalian brain volume with an exponent about 0.85 or 5/6 (Karbowski, 2007, 2011). The important point is that this scaling exponent is significantly larger than a corresponding exponent relating whole body metabolism with body volume, which is 3/4, and is known as the “Kleiber law” (Kleiber, 1947; Schmidt-Nielsen, 1984; West et al., 1997). The implication of this is that as brain increases in mass, its metabolic cost grows faster than the metabolic cost of the rest of the mammalian body. For instance, if brain volume increases 100 times, its energetic cost increases 50 times, whereas a corresponding enlargement of body volume increases the body energetic demand by a substantially smaller factor of 31. This expensive neural energetics can have important consequences for the brain design, i.e., the way evolution has built the mammalian brain and its cortical structure.

### Local scaling of volume-specific cortical metabolism

Given the above scaling for total brain metabolism, it is easy to notice that brain metabolism per volume, i.e., CMR (volume specific cerebral metabolic rate) decreases with brain volume, with an exponent about −0.15 or close to −1/6 (Karbowski, 2007). Interestingly, this volume-specific exponent is also conserved locally across all investigated regions of the cerebral cortex (and most of subcortical structures of gray matter) despite regional heterogeneity of CMR (Karbowski, 2007). This means that the energy utilization of 1 mm^3^ of different cortical areas (e.g., visual and frontal) diminish with increasing brain size in a similar allometric pattern across brains of different species. From this, it follows that 1 mm^3^ of human visual cortex uses about four times less energy than 1 mm^3^ of mouse visual cortex, and similarly for other cortical areas. These facts may suggest a common mechanism of energy utilization in different brain regions that is evolutionary conserved from small to large mammals.

### Metabolic energy per neuron

The conserved mechanism of energy use may have something to do with the question of how energy is distributed globally among neurons. Data analysis of cerebral metabolic rates and neuron density across several mammals reveals that their ratio is almost invariant, both for whole brain and cerebral cortex (Herculano-Houzel, 2011b). This implies that energy utilization per cortical neuron is on average conserved across different species, regardless of their size (Herculano-Houzel, 2011b). This constancy is even more remarkable if one realizes that neurons in larger brains are generally larger, i.e., they have longer axons and dendrites (Braitenberg and Schüz, 1998). Interestingly, neurons in large brains are on average less electrically active than neurons in small brains (Karbowski, 2009), and yet their metabolism is approximately conserved.

### Cortical blood flow per neuron

Cerebral blood flow is strictly related to cerebral metabolism. Generally, the former is a driving force of the latter (Buxton and Frank, 1997; Hyder et al., 1998). The data for several mammalian species show that under regular conditions the average values of these two parameters are linearly correlated (Klein et al., 1986; Noda et al., 2002). Consequently, cerebral blood flow CBF in the cortex scales allometrically with cortical volume with the same exponent as does cortical CMR, which is approximately −1/6 (Karbowski, 2011).

Proportionality of CMR and CBF, together with the above fact that cerebral metabolic rate per neuron density is almost constant (Herculano-Houzel, 2011b), implies that cerebral blood flow should be proportional to neuron density. In other words, a mean blood flow per neuron should be roughly constant as well. Indeed, the data for cortical tissue confirms this constancy of brain circulation (Karbowski, 2011). Typically, there is about 1.45· 10^−8^ mL/min of blood flow per neuron in the cortex (Karbowski, 2011).

### Ratio of CMR/CBF across species and cortical regions

**Figure 4:**
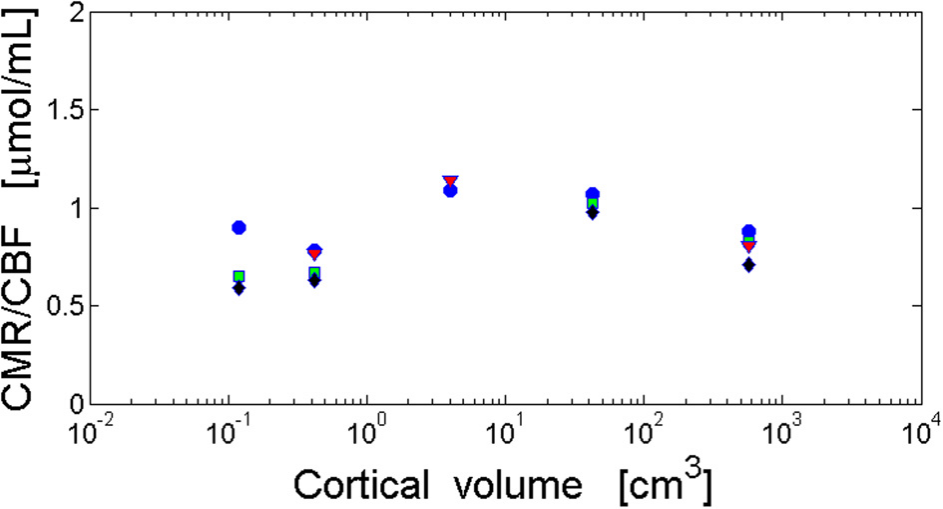
**Invariance of the ratio of cortical metabolism to cortical blood flow (CMR/CBF) with respect to brain size**. The ratio CMR/CBF is independent of cortical volume and cortical area. The log–log fit gives non-significant scaling exponents close to zero. Visual cortex (blue circles): *y* = 0.012x − 0.037 and *r* = 0.288, *p* = 0.638. Frontal cortex (green squares): *y* = 0.041x − 0.14 and *r* = 0.774, *p* = 0.226. Temporal cortex (black diamonds): *y* = 0.036x − 0.17 and *r* = 0.626, *p* = 0.374. Parietal cortex (red triangles): *y* = −0.006x − 0.044 and *r* = −0.094, *p* = 0.940. Data from Table 2.

Conservation of cerebral metabolic energy and blood flow per neuron across mammals (Herculano-Houzel, 2011b; Karbowski, 2011) implies that their ratio should be also brain size independent. This observation is consistent with the data for several mammals (Table 2), i.e., the ratio CMR/CBF is approximately constant in different parts of the cortex (Figure 4). This constancy shows that the cerebral supply of energy and its utilization are strongly coupled and the nature of this coupling is evolutionary conserved.

**Table 2:** **Metabolic and hemodynamic characteristics for mammalian cerebral cortex**.

**Species**	**Visual cortex**	**Frontal cortex**	**Temporal cortex**	**Parietal cortex**
**MOUSE**			
CMR	1.11	1.07	0.93	−
CBF	1.24	1.65	1.57	1.66
CMR/CBF	0.90	0.65	0.59	−
**RAT**			
CMR	0.91	0.83	1.23	0.87
CBF	1.16	1.24	1.95	1.13
CMR/CBF	0.78	0.67	0.63	0.77
**RABBIT**			
CMR	0.76	−	1.02	0.73
CBF	0.70	0.67	−	0.64
CMR/CBF	1.09	−	−	1.14
**MACAQUE**			
CMR	0.63	0.46	0.52	0.47
CBF	0.59	0.45	0.53	−
CMR/CBF	1.07	1.02	0.98	−
**HUMAN**			
CMR	0.38	0.34	0.32	0.35
CBF	0.43	0.41	0.45	0.43
CMR/CBF	0.88	0.83	0.71	0.81

All metabolic data were taken from Karbowski (2007), and all hemodynamic data from Karbowski (2011) (references therein). CMR refers to glucose utilization rate expressed in μmol/(g^*^min), while CBF is cerebral blood flow in mL/(g^*^min).

### Conserved characteristics of cortical capillaries

A combination of theoretical and empirical analysis has provided some clues about a mechanism underlying the above metabolic and hemodynamic invariants (Karbowski, 2011). Energy available in the cortex is constrained by a geometric design of the microvascular system (Blinder et al., 2013). In particular, based on the Krogh model (Krogh, 1929; Boero et al., 1999), oxygen cerebral metabolic rate CMR_*O2*_ is proportional to volume density of capillary length (Karbowski, 2011). The empirical data across adult mammals in cortical gray matter show that capillary length density and neuron density scale similarly with brain size (Karbowski, 2011). This means that both of these parameters are allometrically proportional, from which it follows that CMR_*O2*_ and neuron density are proportional as well. In that way one can phenomenologically explain the constancy of the metabolic energy per neuron that is observed in adult mammals (Herculano-Houzel, 2011b).

The fact that capillary length density is proportional to neuron density implies the constancy of capillary length per cortical neuron among adult mammals (Karbowski, 2011). On average, there is approximately 10 μm of capillaries per cortical neuron (Karbowski, 2011). For human cortex with 2· 10^10^ neurons (Pakkenberg and Gundersen, 1997), this gives about 200 km of capillaries within cortical tissue!

Another invariant associated with capillaries is the portion of cortical space they occupy. Capillary volume comprises about 1–2% of cortical volume, regardless of brain size in adult mammals (Karbowski, 2011). This percentage is more than an order of magnitude smaller than a corresponding figure, ~66%, for a total neuronal wiring (Braitenberg and Schüz, 1998; Chklovskii et al., 2002), which may suggest an economical design of the microvascular system in the cortex.

### Metabolic energy per synapse during development

It has been known for some time that cerebral metabolic rate correlates qualitatively with synaptogenesis (Huttenlocher and Dabholkar, 1997; Chugani, 1998). Synaptic density (Winfield, 1981; Zecevic and Rakic, 1991; Bourgeois and Rakic, 1993; Huttenlocher and Dabholkar, 1997), and glucose metabolic rate (Chugani and Phelps, 1986; Chugani et al., 1991; Chugani, 1998) both exhibit similar and non-monotonic dependence on developmental time, which suggests some coupling between synaptic number and consumed metabolic energy. This simple qualitative observation has recently been generalized and made more quantitative, by finding that during the time course of brain development (from birth to adulthood) the metabolic energy per synapse within a given cortical region is essentially conserved (Karbowski, 2012). More precisely, the metabolic rate per synapse can differ among mammals and cerebral locations, but it is approximately constant during developmental time for a given cortical region of a given species (four species and nine cortical areas investigated). For example, a typical synapse in the macaque monkey and human cerebral cortex consume about 7000 glucose molecules per second (Karbowski, 2012). The regional metabolic constancy during synaptic development suggests an active regulation of cortical metabolic rates that are closely tied to synaptic density. One can speculate that this mechanism is in some sense evolutionary optimized, i.e., each cortical region sets its optimal energy levels for a typical synapse to perform economically its function (see Discussion).

### Distribution of energy use among neural components

How the large amounts of energy consumed by the brain are distributed among neural components, or equivalently, which neural processes are the most energy demanding? Recent study by Hyder et al. (2013) indicate that the ratio of signaling (associated with neural and synaptic activities) and non-signaling (resting activity) components of the cerebral metabolic rate are conserved across different activity levels in rats and humans. The non-signalingpart is about four times smaller than the signaling part. The signaling component can be decomposed into spiking activity and synaptic activity (Attwell and Laughlin, 2001; Karbowski, 2009). Early phenomenological calculations indicated that neural action potentials require more energy than synaptic transmission (Attwell and Laughlin, 2001; Lennie, 2003). However, later experimental studies, using fMRI (Logothetis, 2008) and electrophysiology (Alle et al., 2009), implicated dendrites with their synapses as the major users of energy even when there was no apparent postsynaptic spiking activity. It seems that the likely cause of the discrepancy between theoretical and experimental results was too small probability of neurotransmitter release that was assumed in the theoretical calculations (Attwell and Laughlin, 2001; Lennie, 2003). A recent correction in the phenomenological calculations (Harris et al., 2012) agrees with the idea that synapses cost the most energy. This conclusion is also consistent with theoretical calculations based on developmental data across several mammals, which indicate that synapses utilize more than 50% of CMR not only in adulthood but also during most of the postnatal period of synaptogenesis (Karbowski, 2012).

## Discussion

### Cortical neuroanatomy and metabolism are mutually interrelated as implicated by conservation and correlated variability in their parameters

It is important to stress that cortical metabolism and neuroanatomy must be related because building and maintaining a functional structure requires some level of energy utilization. This simply follows from a physicality of the brain (Laughlin and Sejnowski, 2003; Karbowski, 2007; Niven and Laughlin, 2008; Bullmore and Sporns, 2012). This paper provides an additional support for this notion by showing that there exists conservation in both neuroanatomical (Braitenberg and Schüz, 1998) and energetic global cortical characteristics (Herculano-Houzel, 2011b; Karbowski, 2011, 2012; Hyder et al., 2013). Specifically, cortical metabolism and structure are coupled through inter-relations between metabolic rates, capillaries and glia on one hand, and synapses and neurons on the other. These couplings take place on three different levels: global allometric, regional, and developmental. Each of them is discussed below.

Allometric coupling means that global metabolic (hemodynamic) and microvascular parameters such as CMR, CBF, and capillary length density scale with cortical volume *V* the same way as does neuron volume density, i.e., as *V*^−1/6^, which was discussed in the previous sections (Karbowski, 2011). This implies that there are allometric correlations between metabolism/microvasculature and neuroanatomy across species. From these empirical facts, it follows that metabolic rate, blood flow, and capillary length per neuron are brain size independent, and thus allometrically conserved. Constant is also the average number of synapses per glia, since densities of synapses and glia are invariant with respect to brain size. Thus, there exists a strong structural coupling between the most energetic neuroanatomical elements (synapses) and energy delivery elements (glia) across mammals.

Values of parameters used in allometric scalings are always mean values averaged over different cortical areas. However, usually there is some small local variability in all parameters. Regional coupling between metabolism and neuroanatomy means that regional and laminar variabilities in metabolic/microvascular and structural parameters should be correlated. Data for visual cortex in adult primates (human and macaque monkey) indicate that capillary length density is the largest in the middle layers 4 and 2/3, and the smallest in boundary layers 1 and 5/6 (Bell and Ball, 1985; Weber et al., 2008). This distribution of capillaries correlates qualitatively with laminar variability in synaptic density, which is typically the highest in the middle layers 2/3 and 4, although laminar differences for synapses are less pronounced than those for capillaries (O'Kusky and Colonnier, 1982; Bourgeois and Rakic, 1993; Huttenlocher and Dabholkar, 1997; Scheff et al., 2001). Data for adult rodents are similar. For rat (parietal and visual cortex) and mouse (somatosensory cortex) both capillary length density and synaptic density assume the largest values in the layers 2/3 and 4, and the smallest in the boundary zones (for rat: Bar, 1980; Blue and Parnavelas, 1983; for mouse: DeFelipe et al., 1997; Blinder et al., 2013). Additionally, for mouse cortex there exists an inter-area correlation between neuron density and density of vascular length and its fractional volume (Tsai et al., 2009). Similarly for macaque monkey visual cortex, neuron density as well as basal oxidative metabolism both correlate with vascular length density and with vascular fractional volume (Weber et al., 2008). There is also some evidence regarding glia-microvasculature coupling. Specifically, the density of astrocytes correlates with capillary density across cortical layers in the mouse somatosensory cortex (McCaslin et al., 2011).

Developmental data for different mammals also show that cortical metabolism is coupled to neuroanatomy. In particular, cortical metabolic rate is strongly correlated with synaptic density, such that the ratio CMR/ρ_*s*_ is approximately conserved from birth to adulthood for a given species and cortical area, despite several-fold variabilities in CMR and ρ_*s*_ (Karbowski, 2012). Moreover, data for cat visual cortex indicate that changes in capillary length density closely follow changes in synaptic density during the whole development (Tieman et al., 2004). Specifically, capillary density decreases slightly from juvenile to adult values as synaptogenesis wanes (Tieman et al., 2004).

### How strong is metabolic influence on cortical neuroanatomy?

To what extent do metabolism and hemodynamics constrain cortical design? Is metabolic energy a strong or a weak constraint on the underlying neuroanatomy? This issue is far from resolved and, in some cases, it is unclear what is the direction of causality between metabolic and neuroanatomical changes (e.g., Chetelat et al., 2013). Below we consider these questions.

The traditional view of neuro-hemodynamic coupling has been that although CBF supplies neurons with nutrients and metabolites, their amounts are strictly controlled by neurons depending on neuronal metabolic needs. This suggests an active signaling in one direction from neurons to microvasculature and blood flow (Iadecola, 2004; Attwell et al., 2010). However, there are also some indications that hemodynamics can modulate neural activity and play a role in information processing, which is called hemo-neural hypothesis (Moore and Cao, 2008). According to this hypothesis, the signaling between neurons and blood flow is likely bidirectional. The general theme of this review is somewhat related to the hemo-neural coupling proposed by Moore and Cao (2008), in the sense that hemodynamics can constrain (or modulate) the structure of cortical circuits. However, there are also several key differences. First, the original hemo-neural hypothesis was applied to information processing in sensory systems, which is characterized by time scales of msec to tens of seconds. In the current review, we consider developmental or even evolutionary time scales, which are orders of magnitude slower. Second, energetic or hemodynamic constraints considered here act mainly on neural structure (synaptic and wiring invariants), and to a lesser degree on neural activities as it is in the hemo-neural hypothesis (Moore and Cao, 2008).

Non-monotonic temporal behavior of synaptic density associated with synapses overproduction early in the development and a similar behavior of metabolic rates, discussed above, may suggest that formation of neural connections in the cortex is not overly restricted by global energetic considerations. However, at the single synapse level the issue is more subtle. Because metabolic rate per synapse is approximately conserved from birth to adulthood within a given species and cortical area (Karbowski, 2012), then there must be some regulation of energy expenditure on a typical synapse (connection) during development. Thus, it seems that in this case energy supplied meets cortical functional demands, but at the same time this demand is somehow restricted.

Similarly, some data on cortical structure show that axons connecting remote neurons do not necessarily choose the shortest paths, at least a fraction of them, which means that wiring length and thus metabolic energy consumed is not always minimized (Raj and Chen, 2011). The reason for these axonal deviations as well as for synaptic overproductions is probably that energy is not the only constraint acting on cortical circuits. In fact, these circuits have to perform some functional operations that are facilitated by the underlying connectivity, which require not only energy but also some other resources (see below). Therefore, it seems that metabolism may be a soft constraint on the developing connectivity in the cortex.

On the other hand, it is well known that too small levels of CBF (and energy) cause neuron death in the process known as neurodegeneration (Martin et al., 1994). In adult human, normal CBF is between 0.45–0.60 mL/(g·min) (Erecinska and Silver, 2001). Irreversible neuronal damage occurs if CBF falls permanently below 0.18 ml/(g·min) (Heiss, 1983; Erecinska and Silver, 2001). Thus apparently, there exist a threshold for CBF below which cortical structure does not survive. Additionally, even relatively mild reduction of CBF during development (carotid artery occlusion) can significantly alter neuron density, cortical vascularization, and the pattern of cortico-cortical connectivity (Miller et al., 1993), indicating that metabolism is a serious restriction on the developing neuroanatomy. These developmental abnormalities usually have a negative influence on behavioral and cognitive abilities during adulthood.

Similarly, too high levels of CBF and CMR can also be disadvantageous for cortical circuits. Prolonged neuronal hyperactivity, such as observed during epilepsy, fueled by excessive supply of blood flow can induce processes (e.g., excitotoxicity) leading to neuron damage (Ingvar, 1986; Olney et al., 1986). Although there are some mechanisms preventing a developing brain from seizure-induced brain damage, the adult brain seems to lack them (de Vasconcelos et al., 2002). These observations imply that in extreme situations neurons are unable to control the amount of energy they need, even for their survival. In these cases energy supplied does not meet neural demands because it is either too small or too large in relation to the needs. As a consequence, metabolism and hemodynamics can place a very strong constraint on cortical organization.

Given a possible brain damage associated with epilepsy, one can ask how much energy can be supplied to the cortex? Is there any upper limit? Theoretical arguments indicate that indeed capillary dimensions (capillary length density) set such a limit (Karbowski, 2011). Thus, metabolic rates are not physically attainable above a certain level. Because of that upper limit, and since synapses use a large portion of the overall brain metabolic energy (Harris et al., 2012; Karbowski, 2012), neural connectivity cannot be too dense and must be in some way restricted.

**Figure 5:**
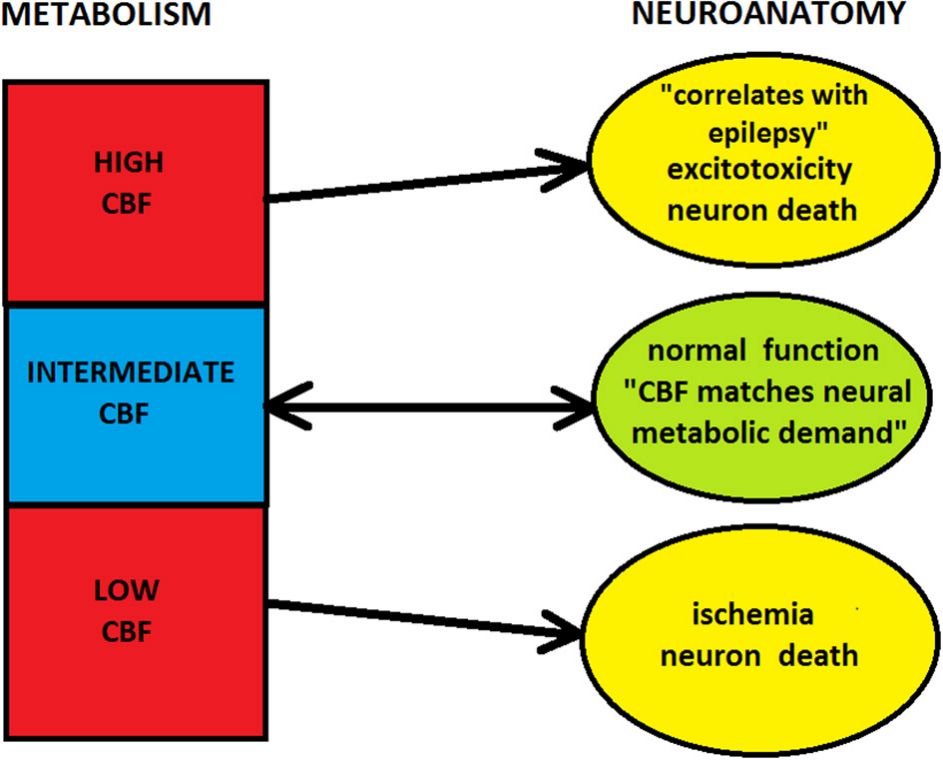
**Influence of cerebral metabolism (hemodynamics) on neuroanatomy**. Too low or too high levels of CBF can lead to the damage of cortical structure. In these regimes blood flow or metabolism strongly constrain neuroanatomy, and neuro-vascular coupling is effectively one-directional, from microvasculature to neurons (i.e., neurons cannot control blood flow). However, for the intermediate level of CBF there is a “window of metabolic opportunity,” where energy supplied by blood flow meets neuronal demands. In this regime, energetic constraint on neuroanatomy is soft, and there is a two-way signaling between microvasculature and neurons (i.e., neurons can control blood flow).

Combining the above considerations, one can note that there likely exist a “metabolic window of opportunity” in the cortex within which energy places a soft influence on neuroanatomy (Figure 5). In this regime, energy supplied seems to meet cortical demands, which is possible due to various signaling pathways from neurons to capillaries (Iadecola, 2004; Attwell et al., 2010). However, outside this window, metabolism places strong constraints on cortical architecture and its neuroanatomical processes. Specifically, too low levels of energy are not viable for functioning, and too large energy amounts promote and sustain pathological conditions, both of which can lead to structural damage if prolonged. Moreover, extremely high metabolic demands are not physically feasible, because of capillary geometric limitations.

### Conserved parameters and optimal brain function

Cerebral cortex has increased its volume and surface area during evolution, but its general neuroanatomical design is essentially conserved across mammals. Many cortical processes associated with connectivity and metabolism look very similar not only qualitatively but also quantitatively from the smallest to the largest species (Tables 1, 2). The fact that some parameters describing the brain are conserved may suggest that they are critical for the cerebral function, and thus may be optimal in some sense. However, we have only a vague clue about how this optimization works, despite several theoretical attempts (Ruppin et al., 1993; Cherniak, 1994; Murre and Sturdy, 1995; Karbowski, 2001, 2003; Chklovskii et al., 2002; Wen and Chklovskii, 2005; Kaiser and Hilgetag, 2006; Bassett et al., 2010). Specifically, we are uncertain about general principles governing cortical structure and function, and how to derive the cortical invariants from these principles. The possibilities range from minimization of temporal delays (Ringo et al., 1994; Chklovskii et al., 2002; Wen and Chklovskii, 2005), minimization of wiring length (Cherniak, 1994; Karbowski, 2001, 2003; Klyachko and Stevens, 2003), minimization of both wiring length and temporal delays (Wen et al., 2009; Budd et al., 2010), minimization of brain volume (Ruppin et al., 1993; Murre and Sturdy, 1995), minimization of both communication path and wiring length (Karbowski, 2001; Kaiser and Hilgetag, 2006), to maximization of information transfer under the energy constraint (Laughlin et al., 1998; Balasubramanian et al., 2001; Perge et al., 2011). It is likely that a single global principle does not exist, and instead cortical (brain) evolution is driven by a combination of “rules”, or evolutionary constraints of different sorts (Kaas, 2000; Laughlin and Sejnowski, 2003; Striedter, 2005). Such constraints often lead to conflicting outputs, which is associated with various trade-offs between structural and functional organization (Karbowski, 2001, 2003; Achard and Bullmore, 2007; Wang et al., 2008; Budd and Kisvarday, 2012; Bullmore and Sporns, 2012). Some of the emerging trade-offs are discussed further.

### Cortical connectivity and allometric scaling

Apart from a general functional role that cortical invariants might play, they also have interesting scaling consequences. As an example, we analyze the scaling of cortical connectivity, both microscopic and macroscopic, with brain size.

Let us start with the microscopic connectivity between neurons. We define an average probability of connection between two neurons as *p* = M/N, where *M* is the average number of synapses per neuron, and *N* is the total number of neurons in the cortex (Karbowski, 2001). An average synaptic density ρ_*s*_ is defined as ρ_*s*_ = NM/V, where *V* is the cortical volume, and an average neuron density ρ_*n*_ is defined as ρ_*n*_ = N/V. Thus, ρ_*s*_/ρ_*n*_ = M and we can write that *p* = ρ_*s*_/(ρ_*n*_*N*) = ρ_*s*_/(ρ^2^_*n*_*V*). Neuron density ρ_*n*_ decreases with cortical volume *V* as ρ_*n*_ ~ *V*^−α^, where the scaling exponent α is generally different for different mammalian orders, and takes values: α = 0.37 for rodents and α = 0.12 for primates (Herculano-Houzel, 2011b). Combining these results with the fact that ρ_*s*_ is scale invariant, we obtain that neural connectivity *p* scales with cortical volume as *p* ~ *V*^2α − 1^. In particular, for rodents we get *p* ~ *V*^−0.26^, while for primates we obtain *p* ~ *V*^−0.76^. These results imply that cortical networks at a microscopic level become sparser as they increase in size. The effect is more pronounced for very large primate brains. For example, for human cortex with *N* ~ 2· 10^10^ (Pakkenberg and Gundersen, 1997), and *M* ~ 3· 10^4^ (DeFelipe et al., 2002) we get *p* ~ 10^−6^, whereas for mouse with *N* ~ 10^7^ and *M* ~ 7· 10^3^ (Braitenberg and Schüz, 1998) we obtain *p* ~ 7· 10^−4^. Thus, the average microscopic connectivity in the human cortex is about 1000 times smaller than that in the mouse cortex.

Interestingly, the number of capillaries per cortical volume and capillary length density also decline allometrically with brain size across mammals, although not that dramatically (Karbowski, 2011). The density of capillary number decreases as *V*^−1/3^, whereas the density of capillary length decreases slightly weaker, as *V*^−1/6^ (Karbowski, 2011). Specifically, between mouse and human these densities fall by factors of 18 and 3–4, respectively (Bell and Ball, 1985; Boero et al., 1999; Blinder et al., 2013). Thus, there is an apparent correlation between the decaying trends in cortical connectivity and capillary densities across mammals, which provides an additional support for a coupling between neuroanatomy and microvascular (metabolic) system.

On a macroscopic level the cerebral cortex is composed of functional areas, each containing many micro modules known as columns. The functional cortical areas have to be somehow connected for an efficient exchange and integration of information (Tononi and Edelman, 1998; Sporns, 2011). We can define the area-to-area cortical connectivity as a fraction of cortical areas “an average” area can connect directly. This connectivity measure was calculated using certain neuroanatomical data, with the conclusion that, at most, it only weakly decays with brain size (Karbowski, 2003). For instance, for mouse cortex the area-to-area connectivity was estimated as 0.30, while for human it was ~0.08, i.e., the human number is only four times smaller (Karbowski, 2003). These theoretical values are similar to the empirical values for cat and macaque monkey, which are respectively 0.27 (Scannell and Young, 1993; Scannell et al., 1995; Young et al., 1995) and 0.15 (Young, 1993; Young et al., 1995), despite large differences in brain sizes between these species. Overall, the probability of macroscopic connectivity is much higher than the probability of microscopic connectivity, which indicates that the nature of neural connections changes from mainly stochastic to mainly deterministic as the scale of description moves form microscopic to macroscopic. Moreover, these two levels of connectivity differ also in terms of allometric scaling, i.e., microscopic connectivity decreases much faster with increasing brain size than macroscopic does.

A related quantity to cortical connectivity is the so-called path length or a degree of cortical separation, which is associated more directly with the efficiency of communication in a network (Karbowski, 2001, 2003; Latora and Marchiori, 2001; Kaiser and Hilgetag, 2006; Achard and Bullmore, 2007). This quantity is defined as a smallest number of intermediate areas (or neurons) one has to visit to connect two arbitrary cortical areas (or neurons) along a given path (Sporns, 2011). It can be calculated either theoretically using some basic neuroanatomical data (Karbowski, 2003), or by analyzing actual connectivity data (Latora and Marchiori, 2001; Kaiser and Hilgetag, 2006). Both approaches yield very similar values of the average path length for cortical areas, around 2.0, which means that on average only one intermediate area is involved in the long-distance communication between two cortical regions. Interestingly, this value is essentially independent of brain size (Karbowski, 2003). The smallness of the cortical path length is one of the two characteristic features of the so-called “small world” networks (Watts and Strogatz, 1998). The other one is modularity or high level of clustering (Sporns, 2011). Both of them occur for the mammalian cerebral cortex (Hilgetag et al., 2000; Sporns et al., 2000), making it a small world network. In this type of architecture, local connectivity is dense, while long-range connectivity is sparser (Perin et al., 2011).

The pattern of cortical connectivity, especially that on the macroscopic level is probably very important for proper cortical functioning. There are many experimental studies suggesting that some mental disorders such as schizophrenia or autism are caused by altered long-range neural connectivity (McGlashan and Hoffman, 2000; Geschwind and Levitt, 2007). Similarly, problems with memory and learning in aged brains associated with Alzheimer disease have been linked to a decline in synaptic density (Rakic et al., 1994; Terry and Katzman, 2001). Taking these facts into account, one may hypothesize that the conserved (or almost conserved) parameters associated with connections, i.e., synaptic density and size, macroscopic connectivity or average cortical path length, are all critical for brain operation and might have been a subject of evolutionary optimization.

### Trade-offs between cortical structure, functionality, and cost

One of the main consequences of the cortical conservation, which is not fully realized and appreciated, is the existence of trade-offs between structural design of the cortex, its cost, and a way the cortex can effectively process and store information. These trade-offs are present on micro-scale in axonal and dendritic arbors design (Wen et al., 2009; Budd et al., 2010; Cuntz et al., 2010; Snider et al., 2010; Teeter and Stevens, 2011), and on macro-scale in fiber pathways configurations (Bassett et al., 2010; Chen et al., 2013). Below, we briefly discuss some of the trade-offs.

#### Connectivity vs. metabolic cost

The analysis above shows that the connectivity between neurons decays quickly with brain size, which generally may not be beneficial for cortical communication because this decaying trend enhances neuronal isolation in bigger brains. To prevent a complete isolation, one would have to increase the length of axons and dendrites to place more synapses on them. However, such an enlargement of neural wiring leads to its larger surface area and volume. This, in turn, is associated with higher influx of Na^+^ ions that have to be pumped out, which costs additional energy. As a result, there exists some compromise between enhancing neural connectivity and reducing the consumption of metabolic energy, which is visible in microscopic and macroscopic patterns of wiring (Bassett et al., 2010; Cuntz et al., 2010; Snider et al., 2010; Teeter and Stevens, 2011; Chen et al., 2013). The connectivity vs. metabolism trade-off is also apparent in a formula for glucose metabolic rate CMR (derived in Karbowski, 2009, 2012), which contains an explicit term proportional to synaptic density. Because of that, one can say that transmission and storage of information in cortical circuits introduces some energy cost. That cost constitutes the majority of the neuronal energy budget (Harris et al., 2012; Karbowski, 2012).

#### Cortical path length vs. metabolic cost

Cortical path length is a measures of neuronal separation. If that separation is large, then the path length is large and interneuronal signal does not reach its target on time. That situation cannot be beneficial for an animal, which often has to respond fast to environmental inputs. Therefore it seems that evolution should keep the cortical path length as small as possible (Karbowski, 2001). However, the path length is negatively correlated with neural wiring, and decreasing the path length leads to the increase in the axon length (Karbowski, 2001; Kaiser and Hilgetag, 2006). This is undesirable, because longer axons mean higher metabolic expenditure. Thus, too short cortical path lengths may cost too much energy (Karbowski, 2001; Kaiser and Hilgetag, 2006).

#### Speed vs. metabolic cost

In a similar fashion, if we want to increase the speed of electric signals traveling along unmyelinated axons in gray matter, we need to increase axon thickness. This is because the conduction velocity of unmyelinated axons is proportional to the square root of axonal diameter (Hursh, 1939; for myelinated axons in white matter this relation is close to linear). Thus, increasing the speed two-fold requires four times thicker axons. With all other things unchanged, this leads to four-fold enhancement of cerebral metabolic rate in gray matter. Again, there is a compromise between increasing the speed of information transfer and saving metabolic energy. The speed of signal transmission along axons is related to temporal delays in the cortex. Generally, these delays should be as small as possible, otherwise the brain would not function coherently (Ringo et al., 1994; Chklovskii et al., 2002; Wen and Chklovskii, 2005; Wen et al., 2009; Budd et al., 2010).

#### Metabolic energy vs. cortical space

Energy available for neurophysiological processes in the cortex is not freely given, but instead it is restricted by the capillary size and glia number. In particular, cerebral metabolic rate is proportional to the fraction of cortical volume occupied by capillary length (Boero et al., 1999; Karbowski, 2011). Thus, for example, increasing energy for synaptic activity (e.g., for storing more memories) would have to be associated with increasing capillary volume and/or glia number in the cortex. But that would implicate less cortical space for other, more functional, neurophysiological processes including synapses, if we are to keep the total cortical volume unchanged. Clearly, energy delivery system needs some space, and this puts a constraint on the amount of possible metabolic rates that are required for cortical efficient function.

#### Neuron size (synaptic weight) vs. neuron activity

How neural spiking activity depends on brain size? Estimates based on cerebral metabolic data show that the average neural firing rate *f* declines with increasing brain size, with an allometric relationship *f* ~ *V*^−0.15^, where *V* is the cortical volume (Karbowski, 2009). The conclusion that lower firing rates are associated with larger brains is in agreement with general observations that physiological processes in larger mammals occur at a slower pace than they do in smaller mammals (Schmidt-Nielsen, 1984). This conclusion is also consistent with allometric data on the decaying trend of firing rates in avian peripheral nervous system (Hempleman et al., 2005).

Moreover, larger brains have neurons with longer fibers (Braitenberg and Schüz, 1998). If we assume that intersynaptic distance along axons and dendrites is invariant (Table 1), we consequently obtain that the number of synapses per neuron is inversely related to neuron's electric activity. Both of these results imply a trade-off between neuron's activity and its synaptic number (and neuron size), i.e., more synapses per neuron actually decrease postsynaptic neuron activity! This trade-off immediately suggests that average synaptic weight should decreases with increasing the number of synapses per neuron (otherwise firing rate would not decrease), or alternatively bigger brains probably have weaker synapses. These conclusions are in line with a computational study, based on developmental data, showing that average synaptic weight declines with firing rate across different mammals (Karbowski, 2012). Moreover, this type of trade-offs (synaptic weights vs. neuronal activity) are characteristic for homeostatic plasticity, which was discovered in slices of cortical circuits (Turrigiano et al., 1998; Turrigiano and Nelson, 2004).

### Limitations of the approach and robustness of invariants

The scaling approach across species taken in this review, as every approach, has its limitations. First, the available empirical data is limited. Therefore, the number of mammalian species used in the scaling plots, and thus the number of data points, is not large. This fact can potentially alter precise bounds of statistical confidence intervals used in describing the scaling trends. Second, each data point represents an average value for a given species. It is good to keep in mind that there is always some degree of variability among individuals of the same species or even across cortical regions of the same individual. Error bars in Table 1 indicate that most variability in our data is in the range 10–20%, and hence it does not seem to be large to affect the robustness of neuroanatomical invariants.

One has to realize that the two above limitations, i.e., small number of species and variability in parameters of interest, may in some cases preclude a clear-cut interpretation of the data. This is the case with surface density of neurons in the cortex, which initially was claimed to be invariant with respect to brain size (Rockel et al., 1980). This constancy was later disputed by others (Herculano-Houzel et al., 2008), and it still causes controversy (Carlo and Stevens, 2013; Young et al., 2013).

### Role of heat released in constraining neuroanatomy

One of the consequences of metabolic processes in neurons is heat generated in the cerebral tissue (Erecinska et al., 2003; Sukstanskii and Yablonskiy, 2006; Kiyatkin, 2007). Since neuronal anatomy and metabolism are mutually related, it is interesting to ask if the heat could have any influence on the underlying cortical structure (Karbowski, 2009)? In particular, how is cortical wiring, i.e., diameters of intracortical axons and dendrites affected? It is known from the laws of thermodynamics that small objects warm up faster than larger ones, and hence one might suspect that axons could warm up excessively due to their small submicrometer diameter. However, this is not the case (Karbowski, 2009). Relatively large cerebral metabolic rates and small fiber diameters are still not enough to warm up the cortical tissue by more than a couple of degrees Celsius (Kiyatkin, 2007). This is mainly due to the circulating cerebral blood that cools the brain in its deeper regions (Karbowski, 2009). Therefore, it appears that temperature in the cortex is almost always well below a critical temperature leading to irreversible damage of neurons and synapses, which is 43–44°C (Karbowski, 2009), provided the environmental temperature is not too excessive. To reach that critical temperature the intracortical wiring would have to be at least ~10 times thinner (Karbowski, 2009). Similarly, the heat in the brain is not large enough to impose a limit on brain size, assuming that both cerebral metabolic rate and blood flow would scale for large hypothetical brains according to expectations given by the allometric scaling (Karbowski, 2009). Concluding, the heat generated in the brain is not the major constraint affecting neuronal wiring and brain size, in spite of previous suggestions (Falk, 1990).

## Concluding remarks

Despite a huge literature concerning cortical neuroanatomy and metabolism, these two subjects have been treated mostly separately in the neuroscience community. A few exceptions in evolutionary and developmental neuroscience dealt with a question of energetic limitations on brain size (Martin, 1981; Aiello and Wheeler, 1995; Isler and van Schaik, 2006; Karbowski, 2007; Navarrete et al., 2011), and with a possible relationship between synaptogenesis and metabolism (Huttenlocher and Dabholkar, 1997; Chugani, 1998; Karbowski, 2012). One of the aims of this review is to show that the mutual relationship between cortical structure and energy is much broader, especially at microscale. This paper discusses several neuroanatomical and metabolic characteristics that are conserved across mammals. Conservation in these two parallel organizations suggests their mutual coupling. In particular, since energy is a driving force of neural organization, and its available amount is always limited, it should constrain some neuroanatomical processes, most notably synapses and neural wiring. However, it is not clear how strong is this restriction. On one hand, there exists an energetic (hemodynamic) threshold below which cortical circuits biophysically degenerate and simply do not function (Martin et al., 1994, Figure 5). On the other hand, for energies supplied above that threshold, the energetic constraint seems to be soft, unless a huge amount of energy is delivered, which may be harmful for cortical structure. The reason for the softness in the intermediate regime is probably that energy cannot be the only evolutionary constraint acting on cortical architecture. Other, perhaps equally important constraints have a functional character and force cortical circuits to perform some functional computations, which requires energy and some other resources.

It is possible that constancy of some of the cortical parameters could be a result of an evolutionary optimization of metabolic, vascular and functional characteristics. However, as was shown above on several examples, any optimization procedure, i.e., maximization and/or minimization of one group of parameters, can lead to a suboptimal performance of another group. Therefore, it is argued that the observed cortical architecture is a result of structural, functional, and energetic compromises, which had to appear during a long evolutionary process.

The importance of metabolic and hemodynamic invariants discussed in this review may go well beyond traditional evolutionary and developmental neurobiology. For example, they can be used in mathematical models of brain activity, which practically neglect any conservation rules (Dayan and Abbott, 2001; Ermentrout and Terman, 2010). Their inclusion could make the models more realistic, and additionally might lead to some non-trivial computational findings regarding structure vs. function relationships. In particular, in models that consider synaptic plasticity and memory phenomena the energy constraint could be implemented. An interesting question would be, for example, what degree of plasticity and how much memory storage is allowed?

### Conflict of interest statement

The author declares that the research was conducted in the absence of any commercial or financial relationships that could be construed as a potential conflict of interest.
